# Breastfeeding effects on DNA methylation in the offspring: A systematic literature review

**DOI:** 10.1371/journal.pone.0173070

**Published:** 2017-03-03

**Authors:** Fernando Pires Hartwig, Christian Loret de Mola, Neil Martin Davies, Cesar Gomes Victora, Caroline L. Relton

**Affiliations:** 1 Postgraduate Programme in Epidemiology, Federal University of Pelotas, Pelotas, Brazil; 2 MRC Integrative Epidemiology Unit, School of Social & Community Medicine, University of Bristol, Bristol, United Kingdom; 3 School of Social and Community Medicine, University of Bristol, United Kingdom; Institut de genomique, FRANCE

## Abstract

**Background:**

Breastfeeding benefits both infants and mothers. Recent research shows long-term health and human capital benefits among individuals who were breastfed. Epigenetic mechanisms have been suggested as potential mediators of the effects of early-life exposures on later health outcomes. We reviewed the literature on the potential effects of breastfeeding on DNA methylation.

**Methods:**

Studies reporting original results and evaluating DNA methylation differences according to breastfeeding/breast milk groups (e.g., ever vs. never comparisons, different categories of breastfeeding duration, etc) were eligible. Six databases were searched simultaneously using Ovid, and the resulting studies were evaluated independently by two reviewers.

**Results:**

Seven eligible studies were identified. Five were conducted in humans. Studies were heterogeneous regarding sample selection, age, target methylation regions, methylation measurement and breastfeeding categorisation. Collectively, the studies suggest that breastfeeding might be negatively associated with promoter methylation of *LEP* (which encodes an anorexigenic hormone), *CDKN2A* (involved in tumour suppression) and *Slc2a4* genes (which encodes an insulin-related glucose transporter) and positively with promoter methylation of the *Nyp* (which encodes an orexigenic neuropeptide) gene, as well as influence global methylation patterns and modulate epigenetic effects of some genetic variants.

**Conclusions:**

The findings from our systematic review are far from conclusive due to the small number of studies and their inherent limitations. Further studies are required to understand the actual potential role of epigenetics in the associations of breastfeeding with later health outcomes. Suggestions for future investigations, focusing on epigenome-wide association studies, are provided.

## Introduction

Breastfeeding has well-established short-term health benefits, and there is increasing evidence that it also has long-term effects on health and human capital [1]. For the effects of an early exposure to persist over time, the exposure must leave some kind of “mark” in the organism [2]. Epigenetics processes–i.e., mitotically heritable events other than changes in DNA sequence that regulate gene expression–have been proposed as important mediators in the developmental origins of health and disease (DOHaD) context [3–5]. Currently, the most frequently studied epigenetic process is DNA methylation, which (in mammals) is the addition of a methyl (–CH_3_) group to DNA at the 5’ position of a cytosine base. In mammals, DNA methylation most commonly occurs in cytosine-guanine (CpG) dinucleotides located in genomic regions called CpG islands–i.e., DNA sequences rich in CpG dinucleotides [6,7].

The notion of epigenetic effects of breastfeeding seems to be widely held, and a Google search (January 23, 2017) using the search terms “epigenetics breastfeeding” resulted in approximately 111,000 hits. There is indeed some evidence supporting the notion that breast milk influences DNA methylation. For example, early-life supplementation of omega-3 fatty acids (an important nutritional compound of breast milk) was associated with methylation profiles in pigs [8]. It has also been hypothesised that the microbiome mediates the effects of breast milk on DNA methylation, since there is evidence that breastfeeding influences the composition of the gut microbiota and that the latter influences DNA methylation [9]. Breast milk also contains long non-coding RNAs [10] and small non-coding RNAs called microRNAs [11], which are involved in gene expression regulation at the post-transcriptional level, suggesting that epigenetic effects of breast milk may not be restricted to DNA methylation.

Three separate literature reviews available to date have suggested the existence of epigenetic effects of breast milk [9,11,12]. However, these reviews were non-systematic and mostly based on evaluations of breast milk properties in isolation rather than comparisons of groups of humans or animals with different feeding modes. We therefore aimed at systematically reviewing the literature on the association between breastfeeding and DNA methylation in humans and animal models.

## Methods

### Search strategy

A systematic review of the literature was performed in August 22, 2016 through Ovid (https://ovidsp.tx.ovid.com/), which allows simultaneously searching of the following databases: MEDLINE, Embase, Allied and Complementary Medicine Database, CAB ABSTRACTS, PsycINFO®, and The Philosopher's Index. By default, Ovid searches the following fields (some of which are database-specific) when all of its databases are searched: Title, Original Title, Title Comment, Abstract, Subject Heading Word, MeSH Subject Headings, Keyword Heading, Keyword Heading Word, Key Concepts, Full Text, Cited Reference Author Word and others.

The following search terms were used for breastfeeding: “breastfe$” OR “breast fe$” OR “bottle fe$” OR “formula fe$” OR “infant feeding” OR “human milk” OR “breast milk” OR “formula milk” OR “weaning”. For epigenetics, the search terms were: “epigenetic$” OR “epigenom$” OR “methylat$” OR “methQTL” OR “mQTL”. Using the wildcard character “$” retrieves any number (including zero) of characters after the stem word (e.g., “breastfe$” retrieves “breastfeeding”, “breastfed”, etc). The two group of search terms were combined using the AND operator: “Breastfeeding” AND “Epigenetics”.

### Study selection and data collection

The aim of our review was to identify studies on DNA methylation differences associated with breastfeeding. Studies were excluded if they met at least one of the following criteria: i) not reporting effects of breastfeeding on DNA methylation (e.g., studies of epigenetic determinants of breastfeeding, such as the association between methylation in promoters of genes involved in breast milk production); ii) being limited to specific breast milk components rather than breastfeeding or breast milk as a whole; iii) not reporting original data.

Eligibility was assessed independently by two reviewers (F.P.H. and C.L.M.), and disagreements were resolved by consensus. Initially, duplicate records were excluded, titles screened and abstracts reviewed. For the remaining studies, full-texts were examined.

The following data were extracted from the included studies:

1. First author’s name and publication year.
2. Country where the study was conducted.
3. Study aim and design.
4. Species, number of individuals, % of females and age.
5. Methylation region, DNA source, measurement method and outcome (e.g., proportion of methylated cells).
6. Breastfeeding categorisation (e.g., never vs. ever, duration in months, etc) and age at ascertainment.
7. Covariates.
8. Breastfeeding-methylation association results.

### Data analysis

Given the lack of consistency between the designs and methods among the studies (as described below), we opted for a narrative review rather than attempting to perform a meta-analysis.

## Results

We evaluated the sensitivity and specificity of our search strategy in a pilot search (S1 Appendix). Briefly, we noted that the Ovid filter to remove non-original publications would likely remove some studies with original data, while the English language filter would likely not substantially influence our study. This pilot search allowed us to reduce the number of publications retrieved in the main search without reducing its sensitivity.

**Fig 1:**
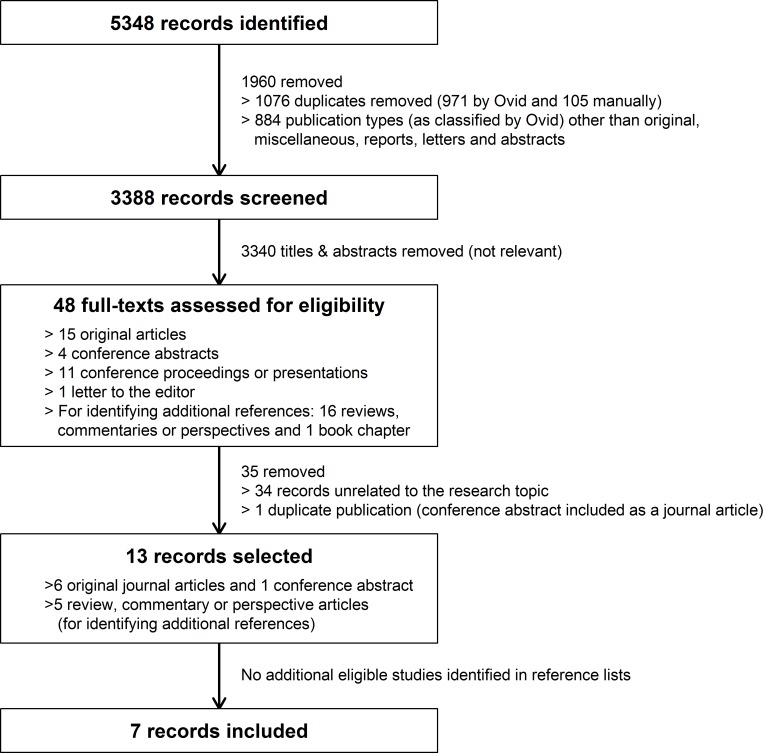
Flow diagram of study selection.

Fig 1 displays a flow diagram of the study selection process. The initial search yielded 5348 records. Of these, 1076 were duplicates. Of the 4272 unique records, 884 were excluded because they were publication types unlikely to include original results according to our pilot search. The remaining 3388 records were screened based on their titles and abstracts, yielding 19 original publications. Another 29 non-original publications were selected only for reference list searching for additional eligible studies, thus totalizing 48 records (S1 Table). After evaluating the full-texts and reference lists, 7 records (6 journal articles and 1 conference abstract) were included (Table 1).

**Table 1:** Characteristics of studies included in the review.

Characteristic	First author, year						
	Obermann-Borst, 2013	Rossnerova, 2013	Soto-Ramirez, 2013	Tao, 2013	Simpkin, 2016	Mahmood, 2013	Raychaudhuri, 2014
**Country**	Netherlands	Czech Republic	England	USA	England^e^	USA	USA
**Study aim**	Evaluate the association of early-life factors with *LEP* promoter methylation in young children	Evaluate if there were methylation differences comparing regions with different levels of air pollution and asthma case/control groups. Other variables (including breastfeeding) were evaluated in secondary analyses	Evaluate potential interactions among genetic variants, CpG sites and breastfeeding, as well as their relationship with asthma	Evaluate the association of early-life factors with methylation in the promoter regions of three genes in breast tumour tissues	Evaluate the association of early-life factors with epigenetic age in children and adolescents.	Compare breast milk with a high-carbohydrate formula regarding epigenetic regulation of *Npy* and *Pomc* genes in the hypothalamus in rats	Compare breast milk with a high-carbohydrate formula regarding epigenetic regulation of *Slc2a4* gene in the skeletal tissue in rats
**Sample characteristics**							
**Species**	Humans	Humans	Humans	Humans	Humans	Rats	Rats
**N**	120	200 (100 asthmatics and 100 controls)	245	639 (all breast cancer cases)	Up to 974	32 (16 per group)	12 (6 per group)
**% females**	42	45	100	100	52	100	0
**Mean age (SD)**	1.4 years (0.2)	11.6 years (2.2)	18.0 years (NA)	57.5 years (11.3)	At birth (NA), 7.5 (0.15) and 17.14 years (1.01)	16 (0) and 100 (0) days	100 days (0)
**Design**	Cross-sectional	Cross-sectional^b^	Longitudinal	Case-case^d^	Longitudinal	Experimental	Experimental
**Methylation**							
**Region**	*LEP* promoter^a^	Global methylation^c^	CpG regions associated with 17q12 genetic variation	*CDH1*, *CDKN2A* and *RARB* promoters	353 CpG sites used to estimate epigenetic age	*Pomc* and *Npy* promoters	*Slc2a4* promoter
**DNA source**	Peripheral blood	Peripheral blood	Peripheral blood	Paraffin-embedded tumour tissue	Cord and peripheral blood	Hypothalamus	Skeletal muscle
**Outcome**	Proportion of methylated DNA copies	Principal component scores of multiple methylated regions	Proportion of methylated DNA copies	Methylation status (yes/no)	Epigenetic age acceleration (regression of epigenetically-predicted age on chronological age), in years	Proportion of methylated DNA copies	Difference of normalised methylation measures^f^
**Measurement**	Mass spectrometry-based quantification of PCR amplicons from bisulfite-converted DNA	Infinium HumanMethylation27 BeadChip	Infinium HumanMethylation450 BeadChip	Methylation-specific qPCR using bisulfite-converted DNA	Infinium HumanMethylation450 BeadChip	Mass spectrometry-based quantification of *in vitro* transcripts generated using PCR amplicons from bisulfite-converted DNA	Southern blot after methylation-sensitive enzymatic cleavage
**Breastfeeding**							
**Categorisation**	Score ranging from 0 to 4, corresponding to 0, >1 –<1, >1–3, >3–6 and >6 months of duration of any breastfeeding, respectively	Duration of full breastfeeding in months	Duration in weeks	0: Ever. 1: Never.	0: Never. 1: Ever.	Breast milk vs. high-carbohydrate milk formula (both from postnatal days 4 to 16 or 24)	Breast milk vs. high-carbohydrate milk formula (both from postnatal days 4 to 24)
**Mean age at ascertainment**	1.4 years	11.6 years	1–2 years	57.5 years	1.0 month	Not applicable	Not applicable
**Covariates**	Bisulfite batch, CpG site, maternal education and smoking at birth, sex, birth weight, current BMI and serum leptin	None	None	Menopause status (stratification), age, education, race and estrogen receptor status	Epigenetic age acceleration was adjusted for cellular heterogeneity	None	None
**Result**	-0.6 (95% CI: -1.19; -0.01) percentage points in methylation per increment in breastfeeding duration category	Pooling asthmatic subjects and controls, breastfeeding was apparently associated with patterns of overall DNA methylation, although no statistical test was performed	There was an interaction between breastfeeding and mQTLs regarding the methylation levels of 10 CpG sites	Odds ratio of *CDKN2A* promoter methylation was 2.75 (95% CI: 1.14; 6.62) times higher in never breastfed women, but only in the premenopausal group (stratification on menopausal status was defined *a priori*)	Pearson’s correlation coefficients (r) and associated P-values (P) were for the association between breastfeeding and epigenetic age acceleration were r = 0.035 and P = 0.301 (at birth), r = -0.010 and P = 0.756 (in childhood), and r = 0.026 and P = 434 (adolescence)	*Nyp* promoter methylation was generally higher in the breast milk compared to the high-carbohydrate formula group. However, there was no strong evidence for methylation differences in the *Pomc* promoter	*Slc2a4* promoter methylation was lower in the breast milk compared to the high-carbohydrate formula group

PCR: polymerase chain reaction. qPCR: quantitative PCR. NA: not available. CpG site: genomic region rich in cytosine-guanine dinucleotides. mQTLs: methylation quantitative trait loci (i.e., genetic variants associated with methylation levels).

^a^Based on seven CpG sites. The outcome for the primary analysis was average methylation across these sites in linear mixed models, although individual-site analyses were also performed.

^b^Even though study participation also depended on asthma case/control status and region, the variables under consideration are methylation and breastfeeding.

^c^Principal component analysis was performed to generate variables that represent global methylation patterns.

^d^The original study was a population-based case-control study, but the analyses involving breastfeeding and methylation were restricted to cases.

^e^Even though Danish and German individuals were also studied in the replication stage, the analyses involving breastfeeding were performed in British individuals only.

^f^Methylation differences were measured using the difference in Southern Blot signal detection between *Hap*II- (blocked by CpG methylation) and *Msp*I- (methylation-insensitive) digested DNA, after normalisation to *Actb* gene.

There were five studies in humans and two in rats, all in high-income countries. Human studies included two cross-sectional studies, two longitudinal studies and one case-only study, with a mean age range of 0 (at birth) to 57.5 years. All studies evaluated distinct and limited genomic regions using six different measurement techniques, although five used methods that involved bisulfite DNA conversion. Four studies analysed blood samples, one analysed paraffin-embedded tumour tissues and the animal studies analysed skeletal muscle and the hypothalamus. Studies also differed regarding breastfeeding categorisation, mean age at ascertainment, selection of covariates and presentation of results.

### Human studies

#### Obermann-Borst et al. (2013)

This was a cross-sectional study in 120 Dutch children (50 girls) at an average age of 1.4 years [13]. The outcome was methylation at the *LEP* gene (which encodes the anorexigenic hormone leptin) promoter in peripheral blood. Methylation was measured using a mass spectrometry-based method involving bisulfite conversion of DNA, yielding the proportion (from 0 to 1) of methylated DNA copies at the sites investigated. In the main analyses, seven different CpG sites in the *LEP* promoter were analysed simultaneously as the outcome variable, using linear mixed models to account for repeated measures. Therefore, the outcome variable can be interpreted as the average methylation in the *LEP* gene promoter as measured by those seven CpG sites. Batch and CpG site were adjusted for in all analyses as fixed effects. Each CpG site was individually evaluated in secondary analysis. Importantly, it is uncertain whether those seven CpG sites, which are within a <170 bp-long region [14], are representative of overall methylation status in this CpG island, which is 625 bp-long and contains 58 CpG sites. Features for this CpG island can be found at the USCS Genome Browser (GRCh38/ hg38 assembly) by searching using the following coordinates: chr7:128,240,698–128,241,322.

Breastfeeding was analysed as a score ranging from 0 to 4, corresponding to 0, >1 –<1, >1–3, >3–6 and >6 months of duration of any breastfeeding, respectively. Information was recorded when the child was 1.4 years old through self-administered questionnaires completed by the mothers. The following characteristics were also evaluated as exposure variables: education, folic acid supplementation and smoking at birth (maternal); sex, birth weight, age, serum leptin levels, growth rate and body mass index (BMI) (children).

In unadjusted analyses, each 1-unit increment in the breastfeeding score was associated with a reduction of 0.6 (95% confidence interval [CI]: 0.01; 1.19) percent points in the proportion of methylated copies of DNA. This corresponded to a relative reduction of 2.9% in DNA methylation. The results were virtually unchanged in analyses adjusting for maternal education and smoking, as well as sex, birth weight, BMI and serum leptin levels of the children. Because child BMI and leptin levels were measured at the average age of 1.4 years, they are not potential confounders of the breastfeeding-methylation association. Indeed, they are potential consequences of *LEP* gene methylation, so adjusting for them might have introduced bias. Nevertheless, it is reassuring that doing so had little effect on the results.

#### Rossnerova et al. (2013)

This Czech study [15] evaluated 200 individuals (mean age of 11.6 years; 89 girls), of whom 100 presented asthma and 100 did not. Half of cases and controls lived in a highly polluted region; the remaining individuals lived in a control region. Case/control status regarding asthma and region were the main exposure variables. Secondary analyses evaluated sex, length of gestation (weeks), birth weight (g), cotinine levels (ng/mg) and length of fully breastfeeding (months).

Methylation was measured in peripheral blood using the Infinium HumanMethylation27 BeadChip, which uses bisulfite DNA conversion and provides the proportion of methylated copies of DNA for approximately 27,800 methylation sites spanning approximately 14,500 genes. This technology has been superseded by a more comprehensive method (described below). For the analysis involving breastfeeding, methylation was evaluated as overall methylation patterns (rather than CpG-site specific analysis) through partial least squares (PLS) with 3 latent factors (although results shown were limited to the 1^st^ and 2^nd^ factors only) and length of gestation, birth weight, cotinine levels and breastfeeding as outcome or response variables. Individuals who were breastfed for longer time had higher values of both factors. Even though this was graphically clear, none of the analyses involving breastfeeding and methylation used statistical tests, which would be essential to evaluate the possible role of chance in the findings.

Furthermore, evaluating the association of breastfeeding with DNA methylation using PLS has some limitations. PLS is not optimal for understanding the relationships between variables. Indeed, the apparently positive relationship of breastfeeding with the PLS factors is difficult to interpret beyond the simple observation that breastfeeding is related to overall patterns of methylation. Second, it is not mentioned in the publication how much of the variation in methylation the 3 PLS factors account for. If this value is low, it is possible that other PLS factors that would account for non-negligible amounts of variation in breastfeeding (which would be indicative of an association between breastfeeding and methylation) might be missed. Analysing the association of breastfeeding with each methylation site individually–a strategy known as epigenome-wide association study (EWAS) [7]–would have provided important and more interpretable biological insights into the potential epigenetic effects of breastfeeding and would have complemented the PLS findings. However, a EWAS in such sample size would likely be underpowered.

#### Soto-Ramirez et al. (2013)

This study (published as a conference abstract) [16] was performed in 245 females participating in the 1989 Isle of Wight Birth Cohort. Peripheral blood methylation data obtained at 18 years of age using the Infinium HumanMethylation450 BeadChip, which provides the proportion of methylated DNA copies for over 485,000 sites, covering 99% of RefSeq (http://www.ncbi.nlm.nih.gov/refseq/) genes. Based on a related publication using this cohort [17] it was possible to identify that breastfeeding was analysed as duration in weeks (probably any breastfeeding, although not specified), ascertained when participants were 1–2 years of age. The overall aim of the study was to evaluate whether there are interactions among breastfeeding, genetic and epigenetic variants with respect to asthma risk. Some important aspects were unclear (possibly due to the brevity of the conference abstract). Following our contact, the authors of the study kindly provided clarifications and additional results, which are described below.

Firstly, eight genetic variants (selected using a linkage disequilibrium filter out of 20 genotyped variants) at the 17q21 locus were tested for association (one at a time) with methylation levels at 26 CpG sites (one at a time) in the same region. The model included the main effects of breastfeeding and genetic variants and an interaction term between these variables. 10 out of the 26 CpGs were influenced by interactions between breastfeeding and genetic variants. This suggests that breastfeeding may modulate the epigenetic effects of some methylation quantitative trait loci (ie, the epigenetic effects of those mQTLs vary according to breastfeeding status). However, it is also possible that some genetic profiles reduce the plasticity of the epigenome, thus mitigating the epigenetic effects of environmental factors. For example, a single nucleotide polymorphism may abrogate a CpG site, thus preventing it from being methylated regardless of the states of other determinants of methylation levels at this specific site. It was not possible to investigate the interaction mechanisms of these associations because neither regression coefficients nor stratified results were available.

Similarly to the study by Rossnerova and colleagues [15], performing an EWAS would have provided important additional biological insights, especially given that the Infinium HumanMethylation450 BeadChip was used, which is the current gold-standard for EWAS in epidemiology studies. Moreover, this study has not been yet published as a full, per-reviewed article, so it must be interpreted in its current form with caution. Study strengths included control of type-I error inflation using the false discovery rate and a relatively short recall period of breastfeeding measurement.

#### Tao et al. (2013)

Tao and colleagues [18] evaluated whether early-life factors are associated with promoter methylation of the *CDH1* (which encodes the cell-adhesion protein cadherin-1), *CDKN2A* (which encodes important tumour suppression proteins such as p14 and p16) and *RARB* (which encodes a receptor for retinoic acid) genes. The analyses involving breastfeeding included 639 women (mean age of 57.5 years) with breast cancer participating in the Western New York Exposures and Breast Cancer Study. Methylation was measured in paraffin-embedded breast tumour tissues using bisulfite-converted DNA followed by methylation-specific quantitative polymerase chain reaction (qPCR). This yielded a binary variable (methylated/unmethylated) for each promoter region. Importantly, since breastfeeding occurred before disease onset, any potential epigenetic effects of breastfeeding would primarily affect healthy cells. Therefore, for associations between breastfeeding and methylation to be detectable in this study, they must still be discernible in tumour tissues. Given that epigenetic dysregulation occurs in many cancers [19,20], it is possible that methylation changes caused by the disease distorted breastfeeding-methylation associations. This issue would have been addressed by analysing paired non-cancerous tissues.

The associations of breastfeeding with promoter methylation were adjusted for age, education, race and estrogen receptor status, and were reported comparing never with ever (reference group) breastfed women. The analyses were also stratified according to menopausal status. In premenopausal women (n = 205), odds ratio estimates were 1.21 (95% CI: 0.50; 2.93), 2.75 (95% CI: 1.14; 6.62) and 1.18 (95% CI: 0.53; 2.62) for *CDH1*, *CDKN2A* and *RARB* promoters, respectively. In postmenopausal women (n = 434), the corresponding estimates were 1.06 (95% CI: 0.64; 1.77), 0.79 (95% CI: 0.49; 1.26) and 1.30 (95% CI: 0.83; 2.04). Analyses were also performed using a composite outcome variable: 1: ≥1 of the three promoters was methylated; 0: none of the promoters was methylated. In these analyses, the odds ratio estimates were 1.87 (95% CI: 0.91; 3.83) and 1.02 (95% CI: 0.67; 1.57) in premenopausal and postmenopausal women, respectively.

Although the above findings suggest that breastfeeding might be related to *CDKN2A* promoter methylation, there were some important limitations. The analyses involved three promoter regions, eight exposure variables, and stratification according to menopausal status. This adds up to 48 comparisons, thus inflating the type-I error rate, which was not corrected. Moreover, although there are conceptual reasons for stratifying according to menopausal status, interaction tests would have been informative regarding whether or not the associations differ between the strata. It is also important to consider that case-control studies involve conditioning on a descendent of the outcome variable. It this study, this is even more pronounced, since it was conditioned on the outcome variable itself. In this situation, associations between breastfeeding and methylation profiles may be biased in different ways, depending on the underlying causal relationships [21]. Therefore, investigating the association between breastfeeding and methylation profiles using other study designs, such as cross-sectional or, ideally, longitudinal studies would be preferred [22].

#### Simpkin et al. (2016)

This study analysed the association between early-life factors with epigenetic age acceleration [23]. The analyses involving breastfeeding (0: never; 1: ever) were performed in up to 974 participants in the Accessible Resource for Integrated Epigenomic Studies (ARIES) project, a sub-study of the Avon Longitudinal Study of Parents and Children [24]. Individuals were epigenotyped using the Infinium HumanMethylation450 BeadChip at birth (cord blood), in childhood and adolescence (peripheral blood). Epigenetic age was estimated using 353 CpG sites applied using the Horvath method [25], and epigenetic age acceleration was computed as the residuals of regressing epigenetic on chronological age. Epigenetic age is an attempt to quantify biological age, and epigenetic age acceleration indicates how much an individual’s epigenetic age is ahead (positive values) or behind (negative values) of his or her chronological age [23].

Breastfeeding was not associated with epigenetic age acceleration at any of the time points investigated in this study, with Pearson’s correlation coefficients (P-values) ranging in magnitude from -0.010 (P = 0.756) to 0.026 (P = 0.434).

The heterogeneity in cell-type composition between cord and peripheral blood (as well as between-individual differences in cell-type composition in the same tissue) could distort associations between breastfeeding and epigenetic clock. In this study [23], epigenetic age was adjusted for cell-type composition estimated using DNA methylation data, as described elsewhere [24,26]. Although measured cell-type composition would be ideal, the estimates used likely at least attenuate any potential confounding. Moreover, Horvath method to estimate epigenetic age is less affected by cell-type composition than Hannum method [27], thus attenuating the possibility of residual confounding eve more. Furthermore, it is possible that this study was underpowered to detect modest effects of breastfeeding on epigenetic age acceleration. This problem could have been attenuated by statistical adjustment for covariates that temporally precede breastfeeding and were associated with epigenetic age acceleration in one or more time points. If those variables are also associated with breastfeeding, this would have also contributed to reducing negative confounding that might exist in the estimates.

### Animal studies

We identified many studies evaluating epigenetic effects of different forms of early-life feeding in animal models, but only two [28,29] comparing breastfeeding with a breast milk substitute.

#### Mahmood et al. (2013)

This study [28] included two groups with sixteen female rats each: one received breast milk and the other received a high-carbohydrate formula. Half the animals in each group were weaned at postnatal day 16 and the other half at day 24, when animals started to receive standard laboratory rodent diet and water *ab libitium*. Epigenetic measures of the promoter regions of the *Pomc* (which encodes a precursor of many peptide hormones) and *Npy* (which encodes the neuropeptide Y) genes promoter were obtained 16 and 100 days after birth in the hypothalamus. Both genes are involved in many physiological processes, including energy homeostasis. Methylation was measured using Sequenom MassARRAY quantitative methylation analysis [30], which yields the proportion of methylated copies of DNA at a specific genomic site.

Rats that received breast milk were shown to display higher methylation in the *Nyp* promoter compared to the high-carbohydrate formula group. They also showed lower levels of *Nyp* mRNA and of histone acetylation (which is another epigenetic marker). Regarding Pomc promoter methylation, there was no strong evidence of a difference. However, the breast milk group presented higher *Pomc* mRNA levels, possibly linked to the higher levels of histone acetylation in this group.

#### Raychaudhuri et al. (2014)

This study [29] design was similar to the aforementioned study,[28] with the following differences: i) all rats were males; ii) there were six rats in each feeding group; iii) weaning occurred at postnatal day 24 only; iv) epigenetic measures were taken 100 days after birth in skeletal muscle tissues. v) the *Slc2a4* gene (which encodes the Glut-4 protein, an insulin-regulated glucose transporter) promoter was evaluated.

Methylation was measured using methylation-sensitive enzymatic cleavage followed by Southern blot. The general idea is to use two enzymes that can cleave the DNA given the presence of specific DNA sequences (called restriction sites). However, the activity of one of such enzymes is blocked if the DNA is methylated, while the other is not. Therefore, DNA fragmentation patterns after enzymatic cleavage depend on methylation. By using a probe that binds to a specific region of the target gene promoter that contains the restriction site, it is possible to measure methylation differences in such promoter. Since the signal was normalised by dividing to a loading control (in this case, the *Actb* gene), the results were in arbitrary units. This form of measurement is semi-quantitative.

Using this strategy, Raychaudhuri and colleagues reported that *Slc2a4* promoter methylation was lower in rats that received breast milk compared to the high-carbohydrate formula group. They also showed higher levels of *Slc2a4* gene expression at both transcriptional and protein levels. Additional evaluations (such as differences in histone acetylation) complemented the results.

Given the experimental nature and the fact that they were performed in an animal model, the two animal studies could evaluate the epigenetic event in the target rather than in a surrogate tissue. They also showed that the observed epigenetic differences were associated with changes in gene expression, suggesting a functional implication of such intervention-mediated epigenetic events.

However, several factors in the two aforementioned animal studies must be considered before extrapolating their findings to humans. First, the purpose of feeding some animals with a high-carbohydrate formula was to evaluate the epigenetic effects of a high-carbohydrate diet in early life, rather than being an attempt to mimic rat milk effects as closely as possible (as in the case of human milk substitutes). This hampers the interpretation of the results, because the epigenetic differences between the two feeding groups could be due to either particular properties of rat milk (e.g., specific nutritional components that have epigenetic effects) or simply the high carbohydrate content in the formula. This issue would have been minimised if it had been an artificial rearing control group fed–i.e., pups artificially fed with rat milk or formula milk that is as similar as possible to rat milk (see below). There was no such group due to the absence of substantial differences between artificial rearing groups fed with a high-carbohydrate formula and with a formula that had a similar caloric distribution to that of rat milk in previous studies [31–33]. However, it may well be the case that the rearing mode is distorting the results because it is well-known that maternal care has epigenetic effects on the offspring [34–37]. Therefore, it is not possible to know if the epigenetic differences between the experimental groups were due to feeding (i.e., high-carbohydrate formula vs. rat milk) or to rearing (i.e., artificial vs. maternal nursing).

## Discussion

Our study summarizes the current evidence regarding the association of breastfeeding with DNA methylation. Collectively, the studies we identified suggest that breastfeeding might be associated with promoter methylation of the *LEP* [13] (negatively) and *CDKN2A* [18] (negatively) genes in humans, and *Npy* [28] (positively) and *Slc2a4* [29] (negatively) genes in rats, as well as implicated in global methylation patterns [15] and in modulation of epigenetic effects of some genetic variants [16]. Moreover, in the *LEP*, *Npy* and *Slc2a4* studies, gene promoter methylation was also associated with higher gene expression levels. This is in agreement with the notion that gene promoter methylation is commonly, although not universally, associated with lower gene expression [38]. Higher gene expression levels of *LEP*, *Pomc* and *Slc2a4* genes and lower levels of the *Npy* gene in breastfed individuals is in agreement with other epidemiological evidence that breastfeeding might protect against obesity and diabetes [1]. *CDKN2A* products have important tumour suppression roles [39] so if breastfeeding really does increase *CDKN2A* expression via epigenetic changes, then it has the potential to protect against cancer. Nevertheless, given the small number of studies and their limitations, it would be premature to make any firm conclusions regarding epigenetic effects of breastfeeding.

In spite of the small number of studies directly addressing the association of breastfeeding with DNA methylation, some authors expressed high expectations regarding these associations (e.g., this commentary [40] and the Google search mentioned above). Although the studies we identified collectively indicate that breastfeeding might be associated with DNA methylation, our systematic review indicates that the evidence is far from compelling and much more research is needed on this topic. Importantly, the present review was focused on DNA methylation changes related to breastfeeding. Future reviews may also address DNA methylation differences due to other foodstuffs or to maternal diets, and to epigenetic changes other than DNA methylation.

In our search we prioritised sensitivity over specificity at the search stage, in order to minimise the possibility of failing to identify eligible studies, which would be particularly relevant in light of the small number of studies on the topic. For this purpose, we searched for studies in many literature databases and piloted our search criteria and filters to avoid excluding eligible studies. The fact that we identified (and included) an eligible abstract and a study that evaluated breastfeeding only in secondary analysis also argues in favour of the sensitivity of our search.

Although our systematic review suggests that breastfeeding might influence DNA methylation, its main conclusion is that more (and better) studies are needed. Particularly, given the focus to date on candidate gene studies or global (non-site specific) measures of methylation, EWAS studies would be very useful to identify regions of the methylome associated with (and possibly influenced by) breastfeeding. Furthermore, these studies must be adequately powered to identify subtle differences in DNA methylation. We used the findings from Obermann-Borst et al. [13] to estimate the sample sizes required to detect DNA methylation differences according to breastfeeding in an EWAS in a total of 18 situations (S2 Appendix and S2 Table). In six of them, up to 1000 individuals were required, suggesting that existing resources (such as the ARIES project) may be properly powered. However, in other scenarios larger sample sizes would be required, and achieving them may be possible through collaborative effort and consortia-based science, examples of which are emerging in the epigenetic literature [41]. Importantly, our calculations are limited because the parameters were obtained from a single study evaluating a single methylation locus with a different method than that used in EWAS.

It is also important that EWAS studies of breastfeeding control for important potential confounding variables. S1 Fig displays postulated causal relationships among breastfeeding, DNA methylation and potential important confounders in the form of a directed acyclic graph [42]. It is well-known that ancestry/ethnicity is an important determinant of indicators of socioeconomic position (e.g., as income, educational attainment, etc) [43–45], and the allele frequencies of many genetic variants are associated with ancestry/ethnicity [46]. Moreover, socioeconomic position is associated with breastfeeding, with the direction of the association differing between income settings [1]. Therefore, if ancestry/ethnicity is associated with genetic variants with direct (i.e., not mediated by breastfeeding) effects on DNA methylation, it may act as a confounder.

Horizontally pleiotropic genetic variants [47] may also confound the association between breastfeeding and DNA methylation. Such horizontal pleiotropy could be mediated, for example, by maternal pre-pregnancy (such as body mass index and parity) and gestational factors (such as maternal smoking during pregnancy, type of delivery and birth weight). This is because epidemiological studies suggest that these factors may influence both breastfeeding [48–54] and epigenetic events [55–61]. Therefore, maternal pre-pregnancy and gestational factors may confound the association between breastfeeding and DNA methylation. Moreover, since family socioeconomic position is associated with those factors [62–65], the latter represent another pathway through which socioeconomic position and ancestry/ethnicity may induce confounding. Another potential pathway is care/stimulation, given that it is associated with family socioeconomic position [66] and, according to studies in animal models, may lead to epigenetic modifications in the offspring. In this context, however, it is important to avoid adjusting for measures of mother-offspring bonding, which may be influenced by breastfeeding [67,68], and therefore mediate (at least partially) its epigenetic effects. Importantly, S1 Fig likely does not exhaust the list of all confounders. We opted by presenting a more parsimonious model focusing one potentially important confounders given the evidence that is currently available. Such model may serve as a basis for more comprehensive models as knowledge on the relationship between breastfeeding and DNA methylation improves.

Another important consideration for future EWAS of breastfeeding is the tissue used to extract DNA. Intra-individual variation (i.e., between tissues of the same individual) in epigenetic patterns is generally higher than variation between individuals [69,70] (although with some exceptions, such as the brain [71]), which limits investigations using easily accessible DNA sources (such as peripheral blood or saliva) when they are not the target tissue [72,73]. This may be an important limitation for epigenetic epidemiology studies of breastfeeding. For example, one of the most strongly supported long-term effects of breastfeeding is its positive association with IQ [74–76]. The optimal DNA source for studying the potential mediating role of DNA methylation in this association would clearly be the brain, but due to practical reasons large-scale epidemiological studies need to rely on easily accessible surrogate tissues. However, some studies suggest that the correlation between epigenetic signatures in the brain and in peripheral blood is generally low, with strong correlations occurring in only a few loci [77–79]. This suggests that, in the case of IQ, the epigenetic studies using DNA extracted from peripheral blood mononuclear cells may provide limited information about DNA methylation in the target tissue. However, this does not mean that such studies are of no utility, since results from some loci would still provide information relevant to the target tissue. Moreover, epidemiological studies suggest that breastfeeding may have long-term effects on other disease outcome, such as obesity and diabetes [1]. More generally, findings from surrogate tissues may provide important insights into the potential range of epigenetic effects of breastfeeding, which may thus inform subsequent studies in tissues of difficult access such as the brain, as well as *in vitro* and *in vivo* studies in animal models. Combining evidence from studies in humans and animals, exploring the strengths of each, is likely to be a fruitful strategy to improve knowledge on the potential epigenetic effects of breastfeeding.

A well-designed and appropriately powered EWAS with good measures of important potential confounders of the association between breastfeeding and DNA methylation would provide important biological insights regarding the well-established associations of breastfeeding with a range of health outcomes [1], as well as to identify potential new biological pathways related to breastfeeding. Moreover, longitudinal DNA methylation data will allow not only identification regions in the methylome associated with breastfeeding, but whether or not such associations persist over time [22,55,61].

Our conclusion is that, in spite of epigenetic mechanisms being postulated by many to explain the links between breastfeeding and long-term outcomes, the literature supporting such claims is remarkably limited. With tempered expectations, adequate definitions and proper research, our understanding of the relationship between breastfeeding and the epigenome will likely improve.

## Supporting information

S1 Appendix Preferred Reporting Items for Systematic Reviews and Meta-Analyses (PRISMA) 2009 checklist. (DOC)

S2 Appendix Pilot literature search. (DOCX)

S3 Appendix Sample size calculations. (DOCX)

S1 Table List of records identified after screening based on titles and abstracts in ascending publication order. (XLSX)

S2 Table Sample size requirements to detect DNA methylation differences according to breastfeeding (ever vs. never) in an epigenome-wide association study (power = 90%; alpha = 2×10^−6^). (DOCX)

S1 Fig Directed acyclic graph depicting postulated causal relationships among breastfeeding, DNA methylation and potential important confounders. U_N_ represents an unknown variable. The thicker line indicates the target causal relationship.
(PDF)
