# Correction: Breastfeeding effects on DNA methylation in the offspring: A systematic literature review

**DOI:** 10.1371/journal.pone.0175604

**Published:** 2017-04-06

**Authors:** Fernando Pires Hartwig, Christian Loret de Mola, Neil Martin Davies, Cesar Gomes Victora, Caroline L. Relton

The following information is missing from the Funding section: CR and ND work in the MRC Integrative Epidemiology Unit, which receives funding from University of Bristol and the UK Medical Research Council (grant MC_UU_12013/2).
