# Supplementary material for: Breastfeeding effects on DNA methylation in the offspring: A systematic literature review
Source: PLoS One. 2017 Mar 3;12(3):e0173070. doi: 10.1371/journal.pone.0173070 (PMC5336253; doi:10.1371/journal.pone.0173070)
Supplement: S2 Appendix — (DOCX) [file pone.0173070.s002.docx]

**Pilot search**

**Methods**

Using an Ovid filter to remove non-original publications, a pilot search was performed using the same search strategy described in the main text in August 27, 2015. We also wanted to evaluate if limiting the search to publications in English would be too restrictive.

4724 records were initially obtained. After removing duplicates in Ovid, 3876 remained (set 1). 2563 remained after removing non-original publications using the Ovid filter (set 2), and 2543 remained after further limiting to publications in English using another Ovid filter (set 3). Not all Ovid databases allow duplicate removal, so potential residual duplicates (classified as such if title and authors’ names were the same) were manually removed. This reduced the number of records to 3806 (set 1), 2543 (set 2) and 2523 (set 3).

The 1263 publications present in set 2, but not in set 1, were classified as “supposedly non-original”. They were distributed (according to the database from which they were retrieved) as follows: 1168 in Journals@OVID, 92 in OVID fulltext Journals@Bristol, and 3 in PsycARTICLES Full Text. 113 supposedly non-original publications (100 randomly sampled from Journals@OVID; 10 randomly sampled from OVID fulltext Journals@Bristol; and all PsycARTICLES Full Text) were analyzed in detail. All 20 publications contained in set 3, but not in set 2, were selected to evaluate the consequences of limiting the search to publications in English only.

**Results**

The distribution of the 113 supposedly (ie, according to Ovid filter) non-original publications (sampled from a total of 1263 studies) according to Ovid classification was: 70 reviews, 21 miscellaneous, 9 editorials, 8 reports, 4 letters and 1 abstract. Of the 21 Ovid-classified miscellaneous publications, 10 were reviews, 5 were original journal article, 1 was an abstract with no original data, 1 was a commentary and 1 was a case report. The remaining 3 were impossible to classify. The only information available for them was their titles: (i) What's in breast milk? A new screening method helps find out (likely a review or a commentary); (ii) IN THIS ISSUE (likely an editorial); (iii) American Journal of Clinical Nutrition: VOL. 70, NO. 4, OCTOBER 1999 (not even a title; possibly an editorial). Although none of the 5 original journal articles were related to the topic of the present review, the fact that there were original publications excluded because they were classified as “miscellaneous” allows the possibility that at least a few relevant studies (not included in this sample) would be excluded by the Ovid filter. Of the 177 miscellaneous publications in the entire list of supposedly non-original publications, 42 (assuming a proportion of 5/21) would be expected to be original publications. Therefore, the main search included miscellaneous publications.

Of the 8 publications classified as reports by Ovid, with 3 being review-like articles, 3 were case reports, 1 was a collection of abstracts (none of them relevant to the topic of the present review) and 1 was an original journal article. Of the 176 publications classified as reports in the entire list of supposedly non-original publications, 22 (assuming a proportion of 1/8) would be expected to be original publications. Therefore, the main search included this publication type.

Of the 4 publications classified as letters by Ovid, 3 presented new data (although none of them was related to the topic of the present review) and 1 of them was a letter to the editor. Of the 29 letters in the entire list of non-original papers, 22 of these (assuming a proportion of 3/4) would be expected to present new data. Therefore, letters were included in the main search.

Regarding language, of the 20 publications in non-English languages according to Ovid, 4 were in Polish, 4 in Hungarian, 4 in French, 2 in Japanese, 1 in Chinese, 1 in German, 1 in Swedish, 1 in Italian, 1 in Spanish and 1 in English (evidencing some lack of specificity in this filter). 9 of them provided new data, but none were relevant to the present review. Therefore, limiting the search to English is not expected to substantially influence the findings from the present systematic review, although it might be important to look for English papers within papers classified as non-English by Ovid. Nevertheless, since the number of publications in languages other than English was small, we opted by not applying a language filter in principle.
