# Supplementary material for: Breastfeeding effects on DNA methylation in the offspring: A systematic literature review
Source: PLoS One. 2017 Mar 3;12(3):e0173070. doi: 10.1371/journal.pone.0173070 (PMC5336253; doi:10.1371/journal.pone.0173070)
Supplement: S3 Appendix — (DOCX) [file pone.0173070.s003.docx]

**Sample size calculations**

Based on the findings by Obermann-Borst and colleagues [[1](#_ENREF_1)], we performed calculations to estimate the sample size requirements to detect DNA methylation patterns associated with breastfeeding in epigenome-wide association studies.

For simplicity, breastfeeding was treated as a binary variable (ever=1; never=0), with prevalence of ever breastfeeding $p\in$ {0.8, 0.9}. The standard deviation of the DNA methylation outcome variable $s$ in the promoter region of the *LEP* gene was 0.3/120 $\cong$3.3 [[1](#_ENREF_1)]. Therefore, we evaluated the following values of s: 1.65, 3.3 and 4.95. The absolute mean change in *LEP* promoter methylation comparing a category with the immediately smaller category was 0.7 percentage points. Given that breastfeeding was treated as a binary variable in our calculations, using 0.7 as the mean difference in DNA methylation comparing ever with never breastfed individuals (denoted by $\beta$) would likely be an underestimation (given that an ever vs. never comparison is much more drastic than a comparison between categories of duration), we used it as the smallest value to be evaluated in the calculations, so that $\beta\in$ {0.7, 1.4 and 2.1}.

Using the Bonferroni correction would yield a statistical significance threshold (alpha level) of 0.05/480,000$\cong$1.4×10^-4^. However, such alpha level is known to be over conservative because it does not account for the correlation between CpG sites. In the study by Richmond et al. [[2](#_ENREF_2)], the false discovery rate cut-off of 0.05 corresponded to a P-value of approximately 2.0×10^-6^, which was then used as the multiple testing-corrected alpha level in our calculations. Power was set to 90%.

**References**

1. Obermann-Borst SA, Eilers PH, Tobi EW, de Jong FH, Slagboom PE, Heijmans BT, et al. Duration of breastfeeding and gender are associated with methylation of the LEPTIN gene in very young children. Pediatr Res. 2013;74:344-349.

2. Richmond RC, Simpkin AJ, Woodward G, Gaunt TR, Lyttleton O, McArdle WL, et al. Prenatal exposure to maternal smoking and offspring DNA methylation across the lifecourse: findings from the Avon Longitudinal Study of Parents and Children (ALSPAC). Hum Mol Genet. 2015;24:2201-2217.
