# Supplementary material for: Breastfeeding effects on DNA methylation in the offspring: A systematic literature review
Source: PLoS One. 2017 Mar 3;12(3):e0173070. doi: 10.1371/journal.pone.0173070 (PMC5336253; doi:10.1371/journal.pone.0173070)
Supplement: S2 Table — (DOCX) [file pone.0173070.s005.docx]

**S2 Table. Sample size requirements to detect DNA methylation differences according to breastfeeding (ever vs. never) in an epigenome-wide association study (power=90%; alpha=2.0×10^-6^).**

| $\boldsymbol{p}$ | $\boldsymbol{s}$ |  | $\boldsymbol{\beta}$ |  |
| --- | --- | --- | --- | --- |
|  |  | **0.7** | **1.4** | **2.1** |
| 0.8 | 1.65 | 1265 | 317 | 142 |
| 0.8 | 3.3 | 5060 | 1265 | 563 |
| 0.8 | 4.95 | 11384 | 2847 | 1265 |
| 0.9 | 1.65 | 2249 | 563 | 250 |
| 0.9 | 3.3 | 8995 | 2249 | 1000 |
| 0.9 | 4.95 | 20237 | 5060 | 2249 |

$p$: Prevalence of ever breastfeeding.

$s$: Standard deviation of the outcome variable.

$\beta$: Mean absolute difference (in percentage points) in DNA methylation between the two breastfeeding groups.
