# Supplementary material for: Breastfeeding effects on DNA methylation in the offspring: A systematic literature review
Source: PLoS One. 2017 Mar 3;12(3):e0173070. doi: 10.1371/journal.pone.0173070 (PMC5336253; doi:10.1371/journal.pone.0173070)
Supplement: S1 Fig — UN represents an unknown variable. The thicker line indicates the target causal relationship. (PDF) [file pone.0173070.s006.pdf]

S1 Fig. Directed acyclic graph depicting postulated causal relationships among breastfeeding, DNA methylation and potential important confounders.

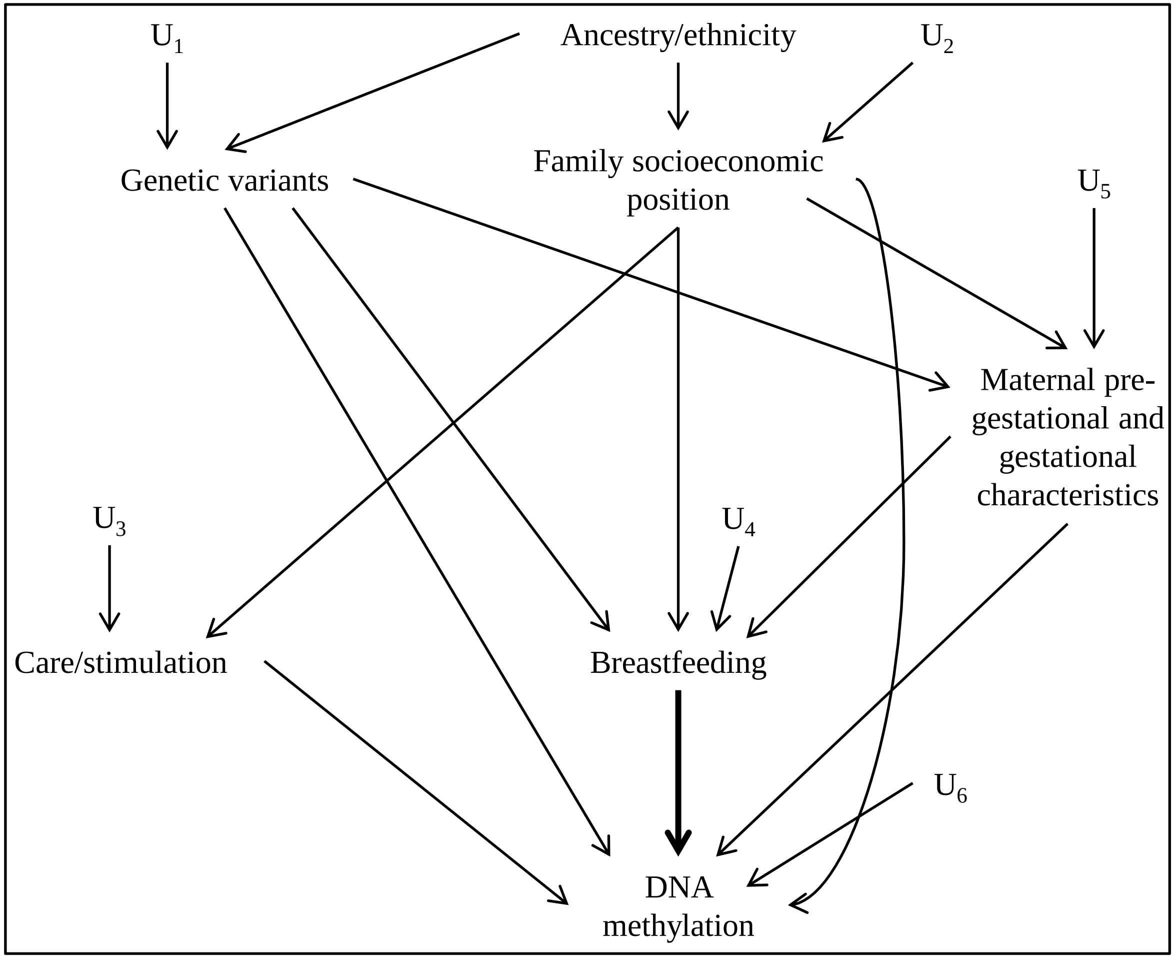

U_N represents an unknown variable.
The thicker line indicates the target causal relationship.
